# Age-dependent carriage of alleles and haplotypes of *Plasmodium falciparum sera5, eba-175,* and *csp* in a region of intense malaria transmission in Uganda

**DOI:** 10.1186/s12936-020-03432-0

**Published:** 2020-10-08

**Authors:** Constance Agwang, Joseph Erume, Brenda Okech, Joseph Olobo, Thomas G. Egwang

**Affiliations:** 1 Med Biotech Laboratories, PO Box 9364, Kampala, Uganda; 2 Makerere University College of Veterinary Medicine, Makerere University, College of Health Sciences, Kampala, Uganda

**Keywords:** Malaria vaccines, Polymorphisms, Immunogenicity, *Plasmodium falciparum*

## Abstract

**Background:**

The development of malaria vaccines is constrained by genetic polymorphisms exhibited by *Plasmodium falciparum* antigens. The project the age-dependent distribution of alleles or haplotypes of three *P. falciparum* malaria vaccine candidates, Circumsporozoite Protein (*csp*), Erythrocyte Binding Antigen 175 (eba-175) and Serine Repeat Antigen 5 (sera5) in a region of intense malaria transmission in Uganda.

**Methods:**

A cross-sectional study was carried out between August and November 2009 in which 250 study participants were selected from a population of 600. Finger prick blood samples were collected after informed consent from participants below 5 years, 5–10 years, and above 10 years of age. Blood was used for microscopy, RDT and dried blood spots. *Plasmodium falciparum* DNA was extracted by chelex method. Alleles of *sera5* and *eba-175* were determined by polymerase chain reaction (PCR) amplification followed by resolution of products by agarose gel electrophoresis. Allele calling was done using gel photographs from ethiduim bromide stained gels. Haplotypes of *csp* were identified by sequencing 63 PCR products using the *P. falciparum* 7G8 laboratory strain sequence as a reference. The data were analysed using SPSS 16, EQX for windows and Chi-square test was used to calculate associations (P-values), Excel was used to generate graphs. The BioEdit and NCBI blast software programs were used to analyse the sequences from which *csp* haplotypes map was constructed.

**Results:**

*Eba-175* FCR3 (48/178) and CAMP (16/178) alleles were observed, the FCR3 (24/67) allele being predominant among children aged below 5 years old while the CAMP (12/67) allele was predominant among older participants. *Sera5* alleles ORI (6/204) and ORII (103/204) were observed in the population, ORII was more prevalent and was significantly associated with age (P values < 0.0001), parasite density (P-value < 0.0001) and clinical outcomes (P value = 0.018). There was marked *csp* diversity in the Th2/Th3 region. Out of 63 sequences, 16 conformed to the reference strain and one (1/16) was similar to a West African haplotype and the majority (14/16) of the haplotypes were unique to this study region. There was an age-dependent distribution of *csp* haplotypes with more haplotypes being harbored by children < 5-year of age, (10/16) compared to adults (2/16). Interestingly, the *csp* haplotype corresponding to 3D7 whose prototypical sequence is identical to the sequence of the leading malaria vaccine candidate RTS, S was not observed.

**Conclusion:**

This data suggest that the *eba-175* FCR3 allele*, sera5* ORII allele*,* and *csp* haplotypes are targets of host immunity and under immune selection pressure in Apac District. These molecules could provide alternative malaria vaccine candidates as sub-unit vaccines**.**

## Background

Malaria is one of the most severe public health problems worldwide. It is a leading cause of morbidity and mortality in many developing countries, where young children and pregnant women are most affected. Nearly half the world’s population lives in areas at risk of malaria transmission in 91 countries and territories. In 2016, malaria caused an estimated 216 million clinical episodes, and 445,000 deaths. An estimated 90% of deaths in 2016 were in the World Health Organization (WHO) African Region [1]^.^ In addition, malaria also causes an unmeasured impact on local economies, human health in general, and longevity [2]. The burden of disease falls mainly on three vulnerable groups: young children under 5 years of age, pregnant women especially the primigravidae in sub-Saharan Africa [1] and persons infected with HIV/AIDS. In Uganda, malaria contributes the major share of the disease burden to the public health system with 93% of the total population at great risk of developing the disease, 25–30% of all outpatient visits, and 35% of in-patient admissions are attributable to malaria alone [3]. There are periodic epidemic outbreaks in South-Western Uganda, while the rest of the country has a stable transmission. Control measures that have been greatly exploited include early diagnosis and prompt treatment, particularly among those at risk of death and severe complications such as young children and pregnant women. Others are improved diagnosis and prophylactic chemotherapy, as well as integrated vector control through the use of insecticide-treated bed nets and indoor residual house spraying [4]. However, over the years, control and treatment was made complicated by the emergence and spread of resistance to insecticides as well as to the inexpensive anti-malarial drugs, chloroquine and sulfadoxine–pyrimethamine [5], which are still used in Uganda for prophylaxis but not as first-line therapies [6]. An effective malaria vaccine will have the potential for the induction of appropriate humoral and cellular immune responses, against several key parasite antigens expressed during the various stages of the parasite life cycle. Each stage in the life cycle provides an opportunity for a vaccine since different proteins are expressed which favor the survival and proliferation of the parasite. Several vaccine candidate antigens are currently under development, these include parasitic and blood-stage antigens [5], such as Circumsporozoite Protein (*csp*), Serine Repeat Antigen 5 (*sera*5), and Erythrocyte Binding Antigen-175 (*eba*-175)*.* CSP, the major component of the most advanced malaria vaccine candidate (RTS,S) is found on the surface of sporozoites. This protein contains two major B cell epitopes consisting of tandem repeats (Asn-Ala-Asn-Pro and Asn-Val-Asn-Pro). Another vaccine candidate that has been considered is based on the *sera*5, also known as the P126 antigen. This is the largest parasite protein, it accumulates in the parasitophorous vacuole of trophozoites and schizonts and is processed into three fragments (18 kDa, 47 kDa, and 50 kDa) [7]. Human clinical trials have established that *sera*5 derived BK-SE36 candidate vaccine is not only safe and immunogenic but protects children against malaria attacks over a year of follow up. Field studies have shown that high titers of IgG anti-SE36 antibodies are associated with protection against malaria in Ugandan children, adults and against placental malaria and low birth weights in pregnant women [8]. *Eba*-175 of *P. falciparum* is localized in the merozoites and is thought to be one of the key players during the fast cascade of interactions between the parasite and host molecules before the merozoites completely invade the erythrocyte [9]. As part of the project’s efforts to prepare for future clinical trials in northern Uganda, the project investigated polymorphisms and allele baseline frequencies of three malaria vaccine candidate antigens *csp, eba-*175*,* and *sera*5. This was to obtain baseline data on these antigen alleles in an area of high malaria endemicity. The study was conducted on the hypothesis that there is no significant association between candidate antigen polymorphisms and participant’s age, clinical presentation/outcomes and parasite density.

## Methods

### Study area

The study was conducted in Apac District, a rural district in Northern Uganda located between Kwania Lake and the Victoria Nile (latitude 1.985; longitude 32.535). Apac District covers an area of 6684 square kilometres and ranges in altitude between 1350 and 1500 m above sea level. The rainfall pattern is bimodal with a dry season from November to February and two short rainy seasons from April to May and from September to October. According to surveys conducted in 2001–2002, this area experiences perennial holoendemic malaria [10], with parasite prevalence rates of 70–90% in children < 10 years of age [10–12]. The entomological inoculation rate was estimated at > 1500 infective bites per person per year and the major vector responsible for transmission is *Anopheles funestus* [10]. *Plasmodium falciparum* is the dominant parasite species, *Plasmodium malariae* being responsible for ~ 3% of the infections and *Plasmodium ovale* was previously not observed [12]

### Study population

The study population comprised of all patients presenting to the health facilities in the selected parishes for clinical management and those who came as a result of mobilization for the study. These were categorized into three age groups of children below 5 years, 5–10, and 10–75 years residing in four selected parishes of Apac district, i.e. Abedi, Atopi, Akere, and Apac parish. Sampling points were health centers for the parishes of Abedi and Akere. For the case of Apac, the main hospital was used as a sampling point. For Atopi, there being no health facility, Atopi primary school was used as a sampling point. Unpublished data (Med Biotech Labs) indicated that each of these target parishes had a unique malaria prevalence among the selected age groups; Apac town residents < 5 years and > 10 years with 68.4% and 16.3%, respectively; Abedi parish < 5 years and > 10 years with 59% and 20.1%, respectively; Akere parish < 5 years and > 10 years with 69% and 20.8%, respectively; whereas Atopi residents < 5 years and > 10 years had 75.9% and 24.1% prevalence, respectively. This data is consistent with findings from other studies which showed that *P. falciparum* malaria in holoendemic areas is characterized by high level of parasitaemia and symptomatic infections in early childhood, and immunity to clinical disease is associated with cumulative exposure [13]. This formed the basis of age categorization into under-five, 5–10 and above 10 years age groups. The study population was further categorized into two categories depending on the parasitaemia. Participants whose parasitaemia was above 5000 parasites/µL of blood and those that had below 5000 parasites/µL of blood. This is consistent with studies in which a dose response model with threshold parameter as a tool for estimation of parasite thresholds and densities showed that children below 5 years had a higher parasite density with an upper threshold for onset of clinical disease was above 5000 parasites/µL, while for 6 years and above had thresholds below 3000 parasites/µL [14]

### Inclusion and exclusion criteria

The inclusion criteria were a positive malaria test for the symptomatic and asymptomatic cases. Those that showed symptoms similar to malaria infection but had negative malaria RDT were excluded from the study. The asymptomatic cases are those who did not present with the cardinal clinical signs for malaria such as high body temperature (above 37.5 °C), general body weakness, headaches and vomiting yet had positive malaria RDT. Symptomatic cases are those who presented with the above signs and also had positive malaria RDT.

### Sampling

Participants were recruited in October 2009 in the four parishes of Apac District. Sampling was done in Apac District Hospital, two health facilities of Abedi and Akere Health Centers II, and a primary school in the parish of Atopi, known as Atopi Primary School. Before the sampling days, community meetings were organized to explain the purpose of the study and to invite people to attend sampling points. At the health facilities and hospital, all clients attending the facilities for clinical care, antenatal visits, or who came specifically to benefit from the screening offered by this study were selected for enrollment. People were sequentially enrolled for the study at all study sites. Written informed consent or in case of illiteracy, consent by thumb printing was obtained from each participant older than 16 years of age and from parents or guardians of younger participants. Each participant enrolled in the study underwent a clinical examination during which an axillary temperature was taken twice the higher of the two was recorded. A single blood sample was obtained by finger prick (approx. 0.3 mL) for thick and thin blood films, for filter paper blood collection (Whattman 3 mm, Maidstone, UK) and Rapid diagnostic tests (RDT; Parachek Orchid Biomedical Systems, Goa, India). This RDT has an estimated detection rate of 97.5% for parasite densities > 2000 parasites/μL and 54.4% for parasite densities of 200/μL.

### Data collection

#### Questionnaire survey

Questionnaires were used for data collection. They were written in English and translated to Luo. Three trained laboratory technologists and nurses were involved in the administration of the individual questionnaires. The questionnaires were first pretested to make sure they elicit the relevant information sought for. Questionnaire target information included demographic data, mosquito net use, type of anti-malarial taken, and when last taken, how long the person had been sick, whether the treatment had been sought and from whom. Interviews were conducted either in English or Luo depending on the subject’s preference.

### Blood sampling

Following questionnaire administration and obtaining consent, finger prick blood was collected from each of the patients. Briefly, the patient’s index finger was cleaned using an alcohol swab, dried with a piece of cotton wool and Vaseline applied onto the cleaned area, this to prevent blood from spreading over the finger. The finger was gently massaged to encourage blood flow to the tip of the finger after which the distal part of the finger was pricked by the use of a sterile lancet. Blood samples were tested using three approaches: (1) blood smears made on microscopic slides for parasite density determination, (2) RDT for rapid malaria diagnosis and, (3) spotting blood onto filter papers for later DNA extraction for molecular characterization of *sera5, eba-175*, and *csp* variants.

### Microscopy

Thick blood films were made from each patient at recruitment. The smears were stained with 3% Giemsa for 30 min. A thick film was considered negative if no parasites were seen in at least 100 high-power fields or if fewer than 500 leucocytes were observed per 100 fields and if reading did not reveal asexual parasites or gametocytes. A slide was considered malaria positive if *P. falciparum* parasites were seen in any of the fields. The thick blood slides were examined later under oil immersion at high magnification (100×) to determine parasite density. Parasite density was calculated from the number of parasites per 200 white blood cells (WBC), assuming an average normal WBC count of 8000/µL.

### Rapid diagnostic testing (RDT)

Rapid diagnostic testing, RDT of malaria infection was carried out to ensure quick treatment administration. The RDT kits were obtained from Orchid Biomedical Systems, Goa, India. The test was carried out following the procedure from the kit given by the manufacturers. Briefly, a finger prick was made on the index finger of the patient and the sample applicator was used to take a loop full of blood. This blood was then applied onto the sample pad, well A and six drops of the lysing buffer was dispensed into well B and the kit was left to stand for 15 min. After which a reading was taken.

### Molecular characterization of *P. falciparum csp, sera5, eba*-175 variants and multiple infections

*Plasmodium falciparum* DNA was extracted from dried blood filter paper samples by the Chelex-100 method, as previously described [14, 15] Briefly, 3 mm pieces of Whattman 3 M filter paper impregnated with dried blood spots were cut and placed in 0.5% saponin overnight at 4 °C. The saponin was removed and replaced with phosphate-buffered saline (PBS) and the tube incubated at 4 °C for another 30 min. The filter paper was removed and transferred into a tube of hot 20%Chelex-100 at 100 °C and maintained at 100 °C for 10 min with intermittent vortexing. After centrifugation, the supernatant containing DNA was used immediately for PCR or stored at − 70 °C until needed. Polymorphic variants encoding the Th2R and Th3R regions of *P. falciparum csp,* N-terminus (octamer repeat domain—OR domain) of *sera5*, and *eba-175* were analysed by nested/semi-nested PCR amplification of parasite DNA as described previously [16–18], For positive controls, genomic DNA from *P. falciparum* HB3, K1 and 3D7 laboratory strains obtained from Malaria Research and Reference Reagent Resource Center (MR4) were used as templates. The details of these procedures are described below. A pair of primers, CSo101 5′-AATCAAGGTAATGGACAAGG-3′ and CSo102 5′-CTAATTAAGGAACAAGAAGG-3′ were used to amplify the full 1193-bp fragment of CSP (GeneDB Accession Number Pf3D7_0304600) in the primary or outer PCR. A second primer pair; CSo101 nucleotides 940–959, 5′-AATCAAGGTAATGGACAAGG-3′ and CSo104 nucleotides 1278–1297 5′-GGAACAAGAAGGATAATACC-3′ was used to amplify the PCR products of the outer PCR in a semi-nested PCR [19]. The PCR mixture contained 5 µL of DNA for both outer and nested PCR in a total volume of 25 µL containing 1 µM concentrations of each primer, 0.2 µM dNTPs, and 0.5 U/25 µL of Taq Polymerase (Roche Diagnostics, Germany) in 1× PCR reaction buffer. PCR amplifications were performed in an MJ Research 96-well thermocycler with the following cycling conditions: Hot start at 95 °C for 4 min, followed by 40 cycles of denaturation at 94 °C (1 min), annealing at 50 °C (1 min), extension at 72 °C (1 min) and a final extension at 72 °C for 4 min. The PCR products were visualized by gel electrophoresis and gel images were digitized and molecular weights assigned to bands using Kodak digital science software. Second-round PCR products were monitored on a 2.0% agarose gel in TAE buffer to check the quality, size, and yield of the PCR products before purification using a Qiagen DNA purification kit. Purified sequence quality DNA was sequenced on contract by Macrogen (South Korea).

A repetitive gene region encoding 3434 bp of the P126 antigen (Gene DB, accession number *Pf3D7*_0207600 was amplified by an outer PCR using primer pairs p126A (5′-AAT GAA GTC ATA TAT TTC CTT G-3′) and p126B (5′-CAA TGT TGT TCT TAA TTC GAT A-3′) followed nested PCR using the primer pairs p126C nucleotides 1–21, (5′-GTG TTA TAT TTA ACA AAA ATG-3′) and p126D, nucleotides 450–471 (5′-CTT ACA GGA TTG CTT GGT TCG-3′) [16]. The PCR mixture contained 5 µL of DNA for both outer and nested experiments in a total volume of 25 µL containing 1 µM concentrations of each primer, 0.2 µM dNTPs and 0.5 U/25 µL of Taq Polymerase in 1× PCR reaction buffer. Both single and nested PCR reactions were carried out using a Gene Amp PCR System 9700 (Applied Biosystem, Foster City, CA) for thirty cycles, consisting of denaturation for 1 min at 94 °C, annealing for 2 min at 47 °C and extension for 3 min at 72 °C. Ten microliter of PCR reaction was loaded onto a 2.5% agarose gel (Sigma, Missouri, USA) in 1× TAE buffer (0.04 M TRIS-acetate, 1 mM EDTA) in the presence of ethidium bromide (0.5 µg/mL). The PCR product bands were visualized under U.V light, photographed, and the presence of bands of 175 bp and 199 bp representing ORI and ORII alleles, respectively, were scored. A 4874-bp PCR product of *eba*-175 (GeneDB accession number *Pf*3D7_0731500) was amplified using primers pairs EBA-1 forward (nucleotides 2336–2356). 5-CAAGAAGCAGTTCCTGAGGAA-3′ and EBA-2 reverse (nucleotides 3060–3083) 5′-TCTCAACATTCATATTAACAATTC-3′ in an outer PCR and primer pairs EBA3 forward nucleotides 2296–2319 5′-GAGGAAAACACTGAAATAGCACAC-3′ and EBA4 reverse (nucleotides 3068–3091) 5′-CAATTCCTCCAGACTGTTGAACAT-3′ for the nested PCR [18–20]. The reaction mixture for both outer and nested PCR consisted of 5 µL of DNA template 0.2 µM of dNTPs, 1 µM of primer, and 0.5 U/25 µL of Taq Polymerase and 1× PCR buffer. Amplification was run in a PTC 100 (MJ research, Inc, USA) thermo cycler under the following conditions: Primary denaturation for 3 min at 95 °C, followed by 30 cycles of secondary denaturation consisting of 1 min at 94 °C, annealing for 1 min at 55 °C, extension for 1 min at 72 °C and final extension of 10 min at 72 °C. Then 15 µL of PCR products were visualized in a 2% agarose gel and the bands scored accordingly. A band size of 795 bp indicated infection with the FCR-3 allele and a band at 714 bp infection with the CAMP allele. The presence of both fragments in one sample indicated a mixed infection of the patient with two different parasite clones.

In order to determine the multiplicity of infection in the study population, the merozoite surface proteins (MSP) were amplified using MSP1 and MSP2 specific primers as described elsewhere [21]. *Plasmodium falciparum* multiplicity of infection might play a role in determining the clinical presentation and outcomes of malaria infections and might contribute to disease transmission rate.

### Data analysis

All data were checked for completeness, coded, and entered in an Excel spreadsheet, transferred to SPSS and analysed. The nucleotide sequence of each *csp* sample was treated as an independent entity. The BioEdit and NCBI blast software programs were used to analyse the sequences from which *csp* haplotype patterns were constructed using 7G8 (MR4) laboratory strain sequence as a reference. Variables were summarized using frequencies and proportions and presented using bar charts. Association between polymorphisms of *csp, eba-175*, and *sera5* on one hand and subject parameters on the other hand were assessed using Pearson's Chi-Square test; P-values of < 0.05 were considered significant.

### Ethical considerations

Approval to conduct this study was granted by Uganda National Council of Science and Technology, Med Biotech Laboratories Institutional Review Board, Makerere University ethics review board and Apac District Administration Board. Information about the study was provided to the participants and informed consent was directly obtained from study subjects above 18 years old. Study subjects considered minors (< 18 years old), informed consent was obtained from the parents or guardians, and assent to the study was directly obtained from the infant. In all cases, the decision of the minor, if possible, took precedence over the consent of the adult parent/guardian. Only consenting adults and assenting minors were recruited into the study.

## Results

### Demographic characteristics of the study population

A total of 250 participants based on the RDT and microscopy results were selected for the study. These were residents of Apac, Atopi, Abedi and Akere parishes, age range from 6 months to 75 years. The males comprised of 41% (106/250) and females comprised 58.7% (144/250). Anti-malarial drug use in the last 2 weeks was reported by 31.6% (79/250) while 68.9% (172/250) reported regular use of insecticide-treated nets (Table 1a). The study population was the categorized into three age groups (< 5, 5–10 and > 10 years old). Apac, Abedi, Atopi and Akere had a total of 49, 68, 70 and 59 participants, respectively. In the age categories of < 5 years: 25, 29, 24 and 22 respectively, 5–10 years olds were; 6, 8, 13 and 29 respectively and in the > 10 year olds; 18, 31, 33 and 29, respectively (Table 1b).

**Table 1:** Demographic information of the study population, panel (a) shows malaria indices and panel (b) shows distribution per age group in the four study sites/parishes

(a) Clinical indices						
	Males (n = 106)			Females (n = 144)		
Temperature > 37.5 n (%)	22 (20.8)			27 (18.75)		
Reported use of antimalarial drug (in 2 weeks), n (%)	47 (44.0)			32 (22.2)		
Reported regular use of bed net, n (%)	72 (67.9)			100 (69.4)		
Reported use of bed net the previous night, n (%)	72 (67.9)			100 (69.4)		

(b) Clinic/Parish and age category distribution						
Parish	Age category					
		1 (< 5)	2 (5–10)	3 (> 10)	Total	P-value (Chi Sq.)
Apac	Count (%)	25 (51)	6 (12.2)	18 (36.7)	49 (100)	0.026
Abedi	Count (%)	29 (42.6)	8 (11.8)	31 (45.6)	68 (100)	< 0.0001
Atopi	Count (%)	24 (34.3)	13 (18.6)	33 (47.1)	70 (100)	0.14
Akere	Count (%)	22 (37.3)	8 (13.6)	29 (49.2)	59 (100)	0.45
Total	Count (%)	100 (40.7)	35 (14.2)	111 (45.1)	246 (100)	

### Malaria indices

**Fig. 1:**
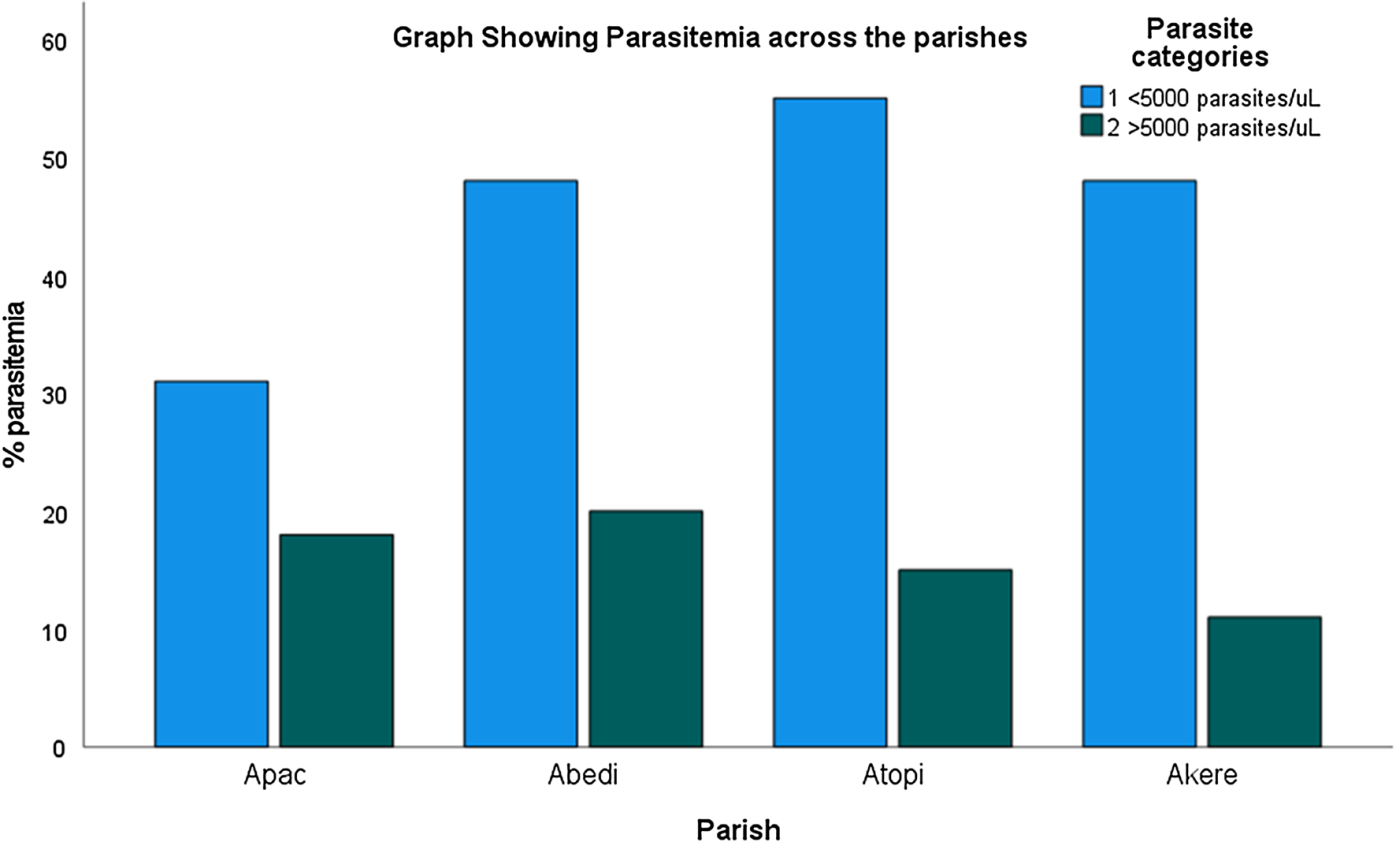
Distribution of parasitemia across parishes. Abedi had the highest percentage of participants with parasitemia > 5000 parasites/µL of blood, while Aere had the least

**Fig. 2:**
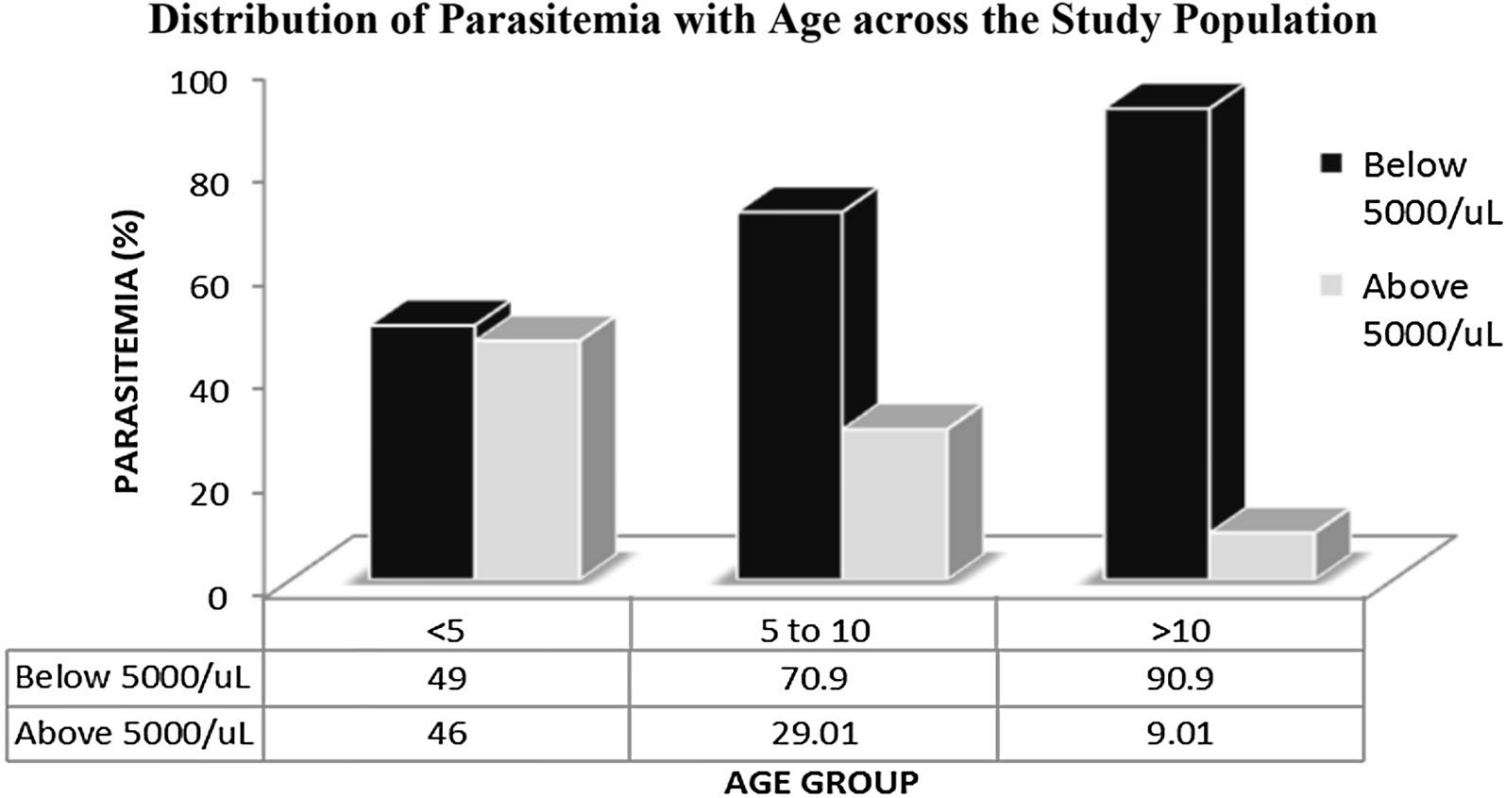
The age dependent distribution of parasitemia in a study population in Apac District, ***a*** region of intense malaria transmission in Uganda. It was observed that parasitemia decreased with age and with density of parasites per microliter of blood. There were more older participants with < 5000 parasites/µL, while younger participants had > 5000 parasites/µL

**Fig. 3:**
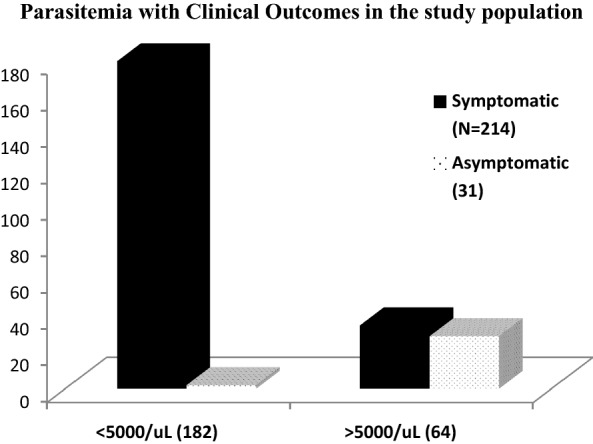
Graph showing distribution of parasitemia with clinicaloutcomes in the study population. There were more participants with parasitemia of < 5000 parasites/µL in the symptomatic group compared to the asymptomatic group. Interestingly, there were more asymptomatic cases in the > 5000 parasites/µL group. This distribution shows that people in Apac district are able to harbour high parasitemia and yet show no symptoms. A function of immunity acquired after repeated exposures

*Plasmodium falciparum* parasitaemia (demonstrable presence of parasites in blood) in the target population was 41.7% (250/600). Only those with a positive malaria test (41.7%) were included in the study, the rest were excluded. The parasitaemia/parasite densities were divided into two categories; < 5000 parasites/µL and > 5000 parasites/µL. This was done to enable determine the difference or any association between parasite density and patient parameters such as age, allele distribution, and clinical presentation/outcomes in all categories. Parasitaemia across the parishes was fairly comparable, although Abedi had the highest number of participants with a parasitaemia of > 5000 parasites/µL and Atopi had the highest number of participants with a parasitaemia < 5000 parasites/µL (Fig. 1). Parasitaemia was also observed to decrease with increasing age, being highest in the < 5 year-olds, intermediate in 5–10-year-olds, and was lowest among the above 10 years and adults (Fig. 2). The association between parasitaemia and age was statistically significant (Chi-square P < 0.0001). Malaria prevalence across parishes was comparable in Apac, Abedi, Atopi and Akere at 20%, 28%, 28% and 24% respectively (Table 2). The project analysed association between clinical outcomes and parasitaemia (Fig. 3); it was observed that clinical presentation/outcomes were significantly associated with parasitaemia, with (Chi square P-value < 0.0001). This is consistent with other data which showed that participants presenting with malaria signs and symptoms often had a high parasite burden in their blood as seen by microscopy and these were mostly falling under the under five group. Further analysis showed that clinical presentation/outcomes were significantly associated with bed net usage, P-value > 0.0001.

**Table 2:** Parasitemia across the three age groups in the study parishes, this table gives a measure of malaria prevalence across the parishes by microscopy

Age category vs parasite categories across Parishes					
Parish	Parasite categories				
	1 (< 5000/µL)	2 (> 5000/µL)	Total	P-value	Parasitemia%
Apac					
Age category					
1 (< 5)	11	14	25		
2 (5–10)	4	2	6	0.011	20
3 (> 10)	16	2	18		
Total	31	18	49		
Abedi					
Age category					
1 (< 5)	13	16	29		
2 (5–10)	5	3	8	< 0.0001	28
3 (> 10)	30	1	31		
Total	48	20	68		
Atopi					
Age category					
1 (< 5)	15	9	24		
2 (5–10)	9	4	13		28
3 (> 10)	31	2	33	0.011	
Total	55	15	70		
Akere					
Age category					
1 (< 5)	14	8	22		
2 (5–10)	7	1	8	0.025	24
3 (> 10)	27	2	29		
Total	48	11	59		100

The statistical association between parasitemia and age groups across the parishes was significant

### SERA5 allele distribution

**Fig. 4:**
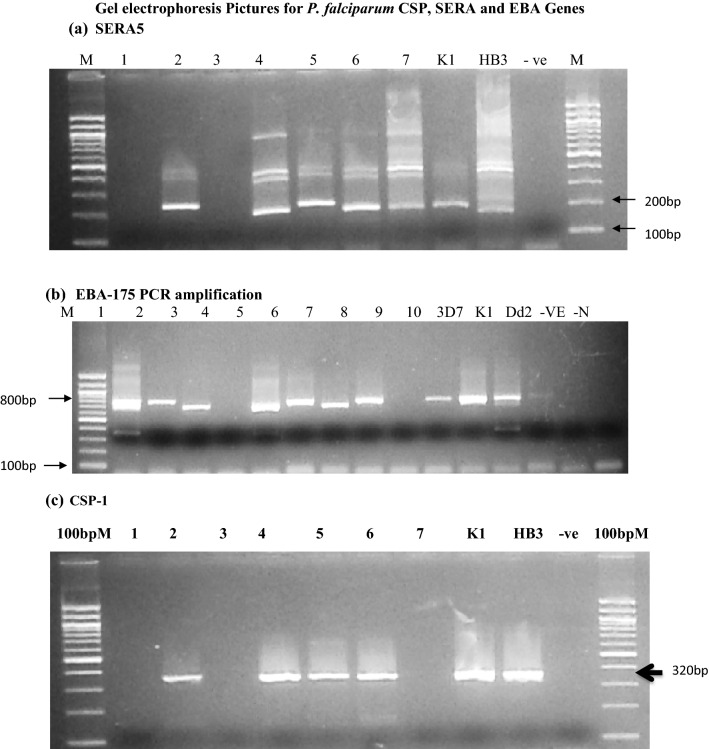
SERA amplification, M is 100 bp molecular weight marker, 1–7 are field samples, K1, HB3 are laboratory isolates of Plasmodium falciparum used as positive controls, −ve is the negative control. The expected product size is at 175 bp (ORI) and 199 bp (ORII). **b** EBA-175 amplification, M, 100 bp molecular weight marker, lane 1 and K1-mixed infections with both 795 bp and 714 bp alleles. Lanes 2, 6, 8, 10, Dd2 and 3D7 infection with the F-allele at 795 bp, while lanes 3, 5 and 7 represent the C-allele at 714 bp. −VE and −N are the negative and nested negative controls. **c** A gel picture of CSP amplification, M, 100 bp molecular weight marker, lanes 1–7 are field isolates, K1 and 3D7 are positive controls, −ve is a negative control. The expected product size is at 321 bp

**Fig. 5:**
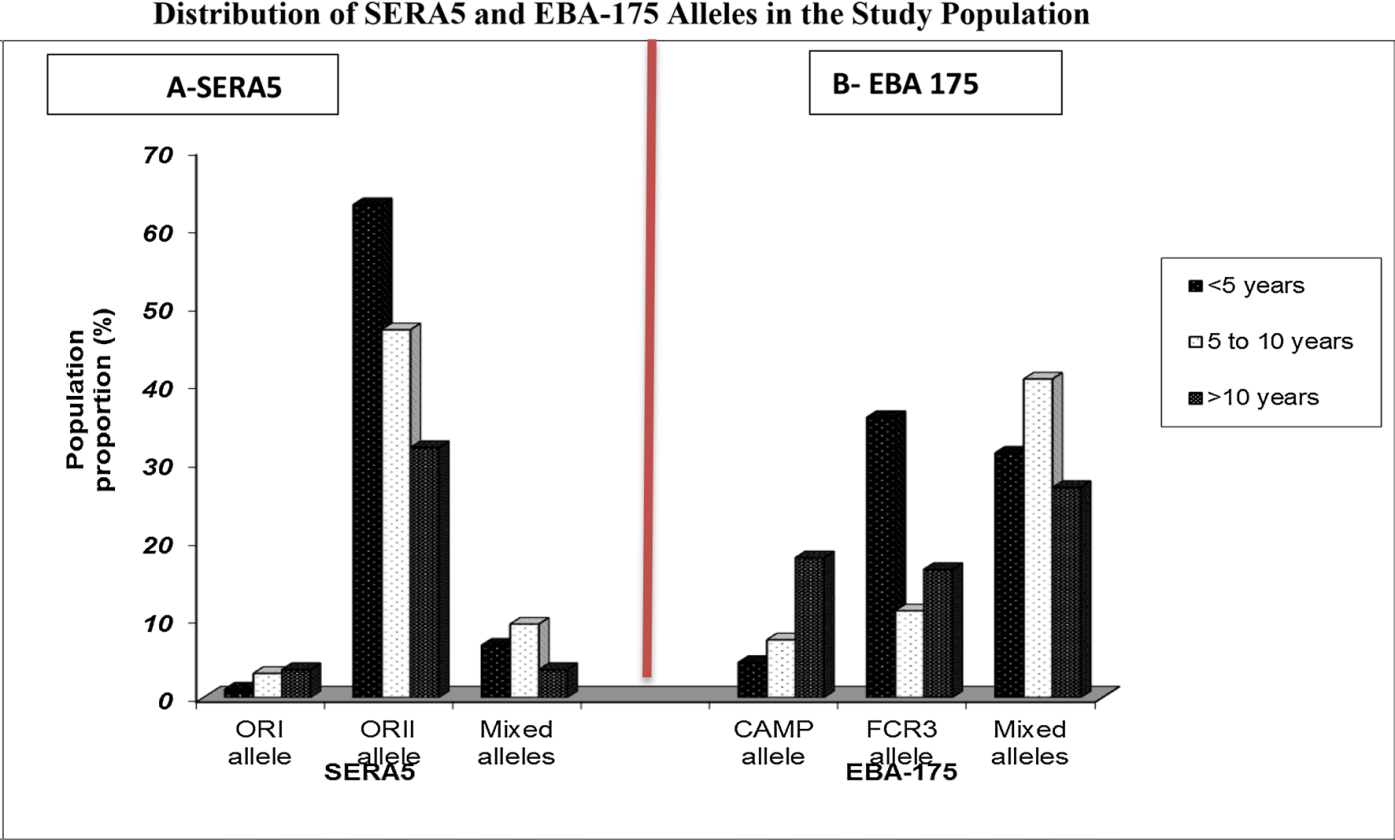
The age dependent distribution of SERA5 (**a**) and EBA-175 (**b**) alleles in a study population in Apac district, a region of intense malaria transmission in Uganda ORII was more prevalent among the < 5 year participants as compared to the those > 10 years. Similarly, FCR fragment was more prevalent in the under 5 population. This could mean that these two figments from different genes are a target of immunity

**Fig. 6:**
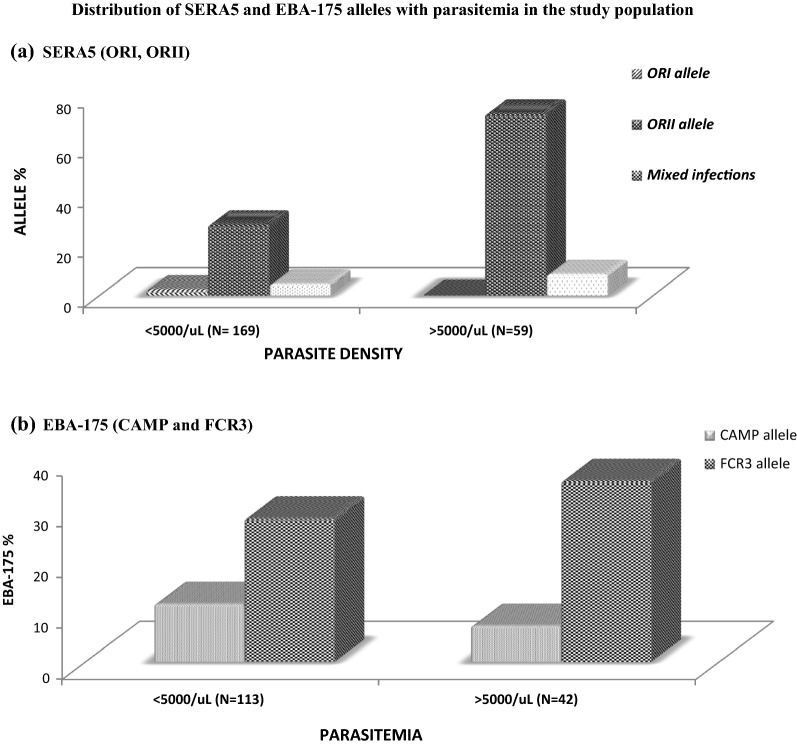
**a** SERA5, ORII allele was more prevalent among participants with a parasitemia > 5000 parasites/µL of blood, while CAMP allele was higher in the group with a parasitemia of < 5000 parasites/µL of blood, **b** EBA-175 FCR allele was observed more in the group with a parasitemia of > 5000 parasites/µL of blood, compared to ORI which was observed more in the group with < 5000 parasites/µL of blood

**Fig. 7:**
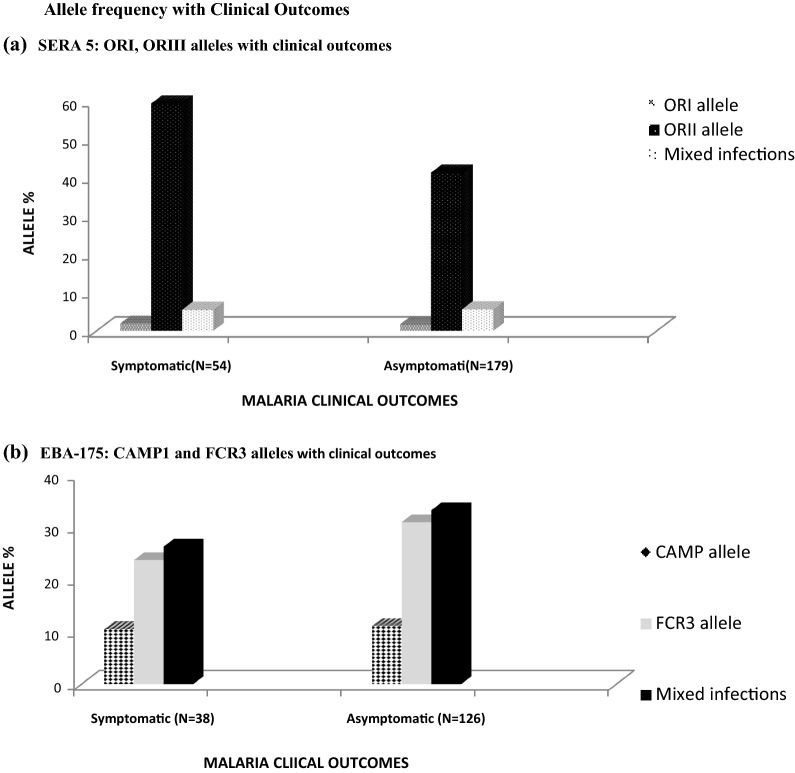
**a** Shows that allele ORII was more prevalent in the symptomatic group compared to the asymptomatic group, **b** shows distribution of EBA-175 alleles with clinical outcomes in the study population. The FCR allele was more prevalent in the symptomatic group compared to the asymptomatic group

Two *sera5* alleles, ORI (175 bp) and ORII (199 bp) were identified by nested PCR analysis in the study region (Fig. 4a). The ORII allele predominated in all the parishes of Apac, Abedi, Akere, and Atopi with a prevalence of 57.0%, 39.1%, 37.0%, and 37.3%, respectively, while the ORI allele was less common with a prevalence of 4.3%, 0%, 1.4%, and 3.4%, respectively. The mixed allele was also less common with Abedi having the highest proportion of 8.7% and Atopi with the lowest at 2.8%. The distribution of the two alleles was investigated in different age groups. The distribution of the ORII allele inversely correlated with age (Fig. 5a). It was highest in the < 5-year-olds with a frequency of 63.0% and lowest among the > 10-year-olds with a frequency of 32.0% while the 5–10-year-olds had an intermediate frequency of 47.0%. The *ORI* prevalence was, however, lower, by comparison, being prevalent at 1.1%, 3.1%, and 3.6% in the < 5-year olds, 5–10-year olds, and < 10-year olds, respectively. The mixed allele (ORI plus ORII) distribution was highest in the 5–10-year-olds. Overall, there was a significant association between *sera5* allele distributions and age (P < 0.0001). Individual analyses of the distribution of alleles with age category presented a divergent statistical picture with ORI*,* ORII, and ORI/ORII showing P values of 0.466, 0.002, and 0.476, respectively. This shows that only ORII was significantly associated with age. ORII allele frequency was significantly higher with a frequency of 11/19 (57.9%) in participants with > 5000 parasites/µL, while the *ORI* allele was significantly lower with a frequency of 6/169 (2.4%) in the same participants (Fig. 6a). Allele distribution was significantly associated with parasite density with P-value, (P < 0.0001). The allele frequency association with clinical outcomes were also statistically significant (P = 0.018). There was a significantly higher prevalence of the ORII allele in the symptomatic group (83/179, 46.4%) by comparison with the asymptomatic group (20/54, 37.0%) (Fig. 7a). There was no significant association between allele distribution on one hand and bed net use (P = 0.855) or gender (P = 0.064) on the other hand.

### EBA-175 allele distribution

Two *eba-175* alleles, CAMP (C-allele) and FCR3 (F-allele) were identified by nested PCR analysis and the FCR allele predominated with an overall percentage of 63.3% in the study population (Fig. 4b). Distribution within the age groups was 35.8%, 11.1%, and 16.4% among the under 5, 5–10, and above 10-year-olds, respectively. For the case of the CAMP allele, the overall percentage was 29.8% in the study population. Distribution within the age groups was: (6/67) 4.5%, 7.4% and 17.9% among the under 5, 5–10, and above 10-year-olds respectively, in the study population. The *eba-175* allele distribution was age-dependent (Fig. 5b). The association between *eba-175* alleles and age were statistically significant (P = 0.035). With regards to clinical outcomes, the FCR3 allele was more frequent (43/135, 31.9%) in the asymptomatic group while the CAMP allele was more frequent in the symptomatic group. However, the association between *eba-175* allele distribution and clinical outcomes was not significant (P = 0.854). The *eba-175* FCR3 allele was less frequent (32/113, 11.5%) in > 5000 parasites/µL was and more frequent (5/12, 41.7%) in the < 5000 parasites/µL group. The CAMP allele, on the other hand, was more frequent in the lower parasitaemia group, that is the < 5000 parasites/µL group (Fig. 6b). Again, there was no significant association between *eba-175* allele distribution and parasite density (P = 0.775). There was also no significant association between *eba-175* allele distribution on one hand, and bed net use (P = 0.555) and gender (P = 0.33) on the other. The project further analysed gender, bed net use and found that there were significant associations with allele distribution in both groups.

### CSP haplotype distribution

Circumsporozoite protein gene, *csp* extracted from *P. falciparum* field isolates was amplified by PCR (Fig. 4c) and the products were sequenced and amino acid sequences were blasted against the *P. falciparum* 7G8 strain sequence which was used as a reference strain. Out of 63 sequenced isolates, 16 isolates and the 3D7 laboratory strain had the *P. falciparum* 7G8 haplotype while 42 isolates and the laboratory strain K1 did not. However, at the nucleotide level, 39 of these 42 sequences matched the 7G8 strain whilst 3 did not. The authenticity of the sequences was further validated by the fact that the sequence of the amplified 3D7 positive control was identical to the 3D7 sequence in the Plasmo DB DNA database.

**Fig. 8:**
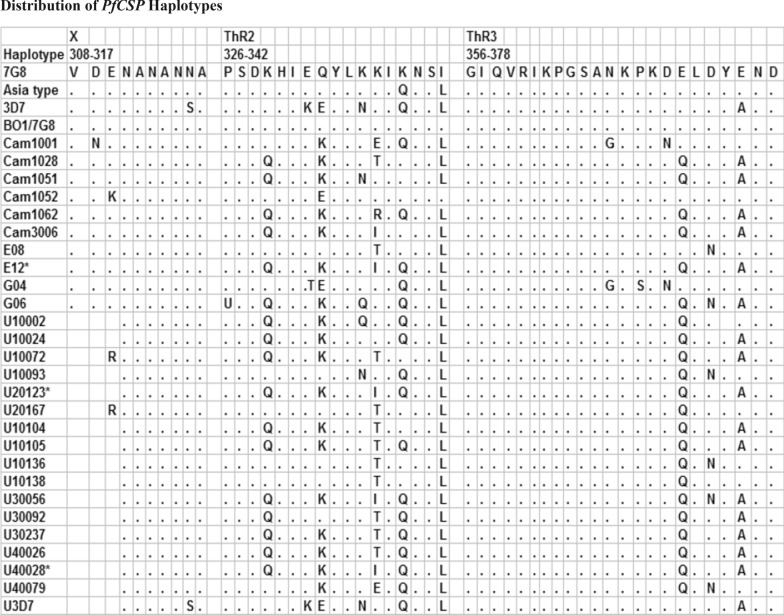
CSP haplotypes observed in Ugandan, Sierra Leonean, Asian, and Gabonese *P. falciparum* isolates. X- Represents the variable site immediately after the central NANP repeats (amino acid residues 308–317). Th2R and Th3R are the non-variable regions in the C-terminal portion of the CSP gene (amino acid residues 326–342 and 356–378 respectively). Haplotype E12, observed in Sierra Leone was similar to one haplotype from two Ugandan samples (U40028 and U20123); these haplotypes are asterisked

Furthermore, all the Ugandan isolates did not have the VDE (valine, aspartic acid, and glutamic acid, respectively) amino acids at region X (amino acids 308–317). Only two sequences had R (arginine) instead of E (glutamic acid) at region 308–317. While 12.5% (2/16) of the haplotypes were found in the adults, 62.5% (10/16) were found in children < 5 years and 25% (4/16) were found in the 5–10-year olds. There was no significant association between age and CSP haplotypes (P = 0.091). Haplotypes frequencies were unequally distributed among the four parishes studied with Akere having the highest number of haplotypes 37.5% (6/16) while Apac and Atopi had the same frequency, 31.3% (5/16). Seventy-five percent (12/16) of the haplotypes were observed in patients with symptomatic malaria whereas 25% (4/16) were observed in those with asymptomatic malaria. Statistically, there was no significant association between clinical presentation and haplotype distribution, (P > 0.5). However, 69.0% (11/16) of haplotypes were in those with > 5000 parasite/μL while 31.0% (5/16) were in those with < 5000 parasite/μL. Very importantly, out of 17 haplotypes observed in the Ugandan population, only two (U20123 and U40028) were similar to one haplotype (E12) observed in Sierra Leone and the rest were unique to the Ugandan study population (Fig. 8).

### Multiplicity of infection

**Fig. 9:**
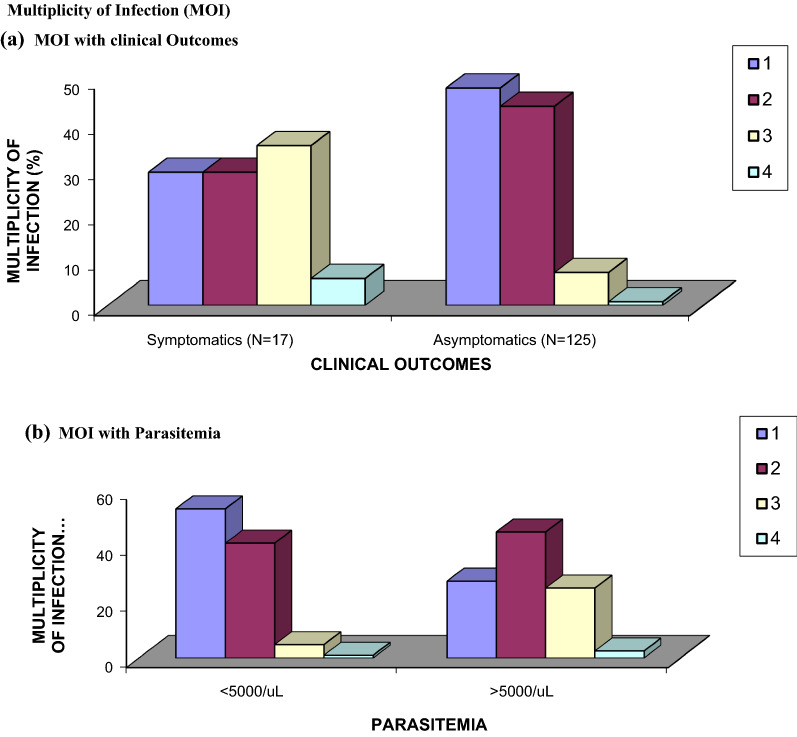
MOI with clinical outcomes (**a**) and parasitemia (**b**). In **a**, MOI was high in the symptomatic group as compared to the asymptomatic group. In **b**, MOI increased with parasitemia

Generally, there were fewer participants with 3–4 infecting parasites in all the parishes. The number of infecting parasite clones in the study population decreased with age, the Chi square test showed a significant association between MOI and age; P-value, 0.01. More parasite clones were also observed in the symptomatic malaria group compared to the asymptomatic malaria group, statistical analysis showed a significant relationship between MOI and clinical outcomes within the study population with a P-value; 0.002 (Fig. 9a). Furthermore, parasite clones increased with parasite density and a statistical analysis showed a significant association between MO and parasitaemia, with a P-value; 0.001 (Fig. 9b).

## Discussion

*Plasmodium falciparum* exhibits considerable genetic diversity, especially among surface antigens that are constantly under immune selection pressure. These surface antigens have also historically been considered as the main targets of immunity [22]. This extreme genetic diversity, however, poses a major challenge to vaccine development. This is because it could lead to vaccine-resistant malaria with non-vaccine type parasites being selected for by vaccine-induced immune pressure [22]. It was of interest to establish the baseline genotypes of *P. falciparum,* which circulate in the study area in anticipation of the future deployment of selected malaria vaccines.

This study aimed to (1) investigate the polymorphism, distribution, and frequency of three major malaria vaccine candidate antigen alleles (*sera, eba-175,* and *csp*) in Apac District; (2) to demonstrate presence or absence of associations between polymorphisms, age, parasite density, and clinical presentations/outcomes.

On the release of SERA5 from the parasitophorous vacuole, it is processed into two fragments of 50 and 73 kD. The 73 kD is composed of two peptides of molecular weight 47 and 18 kD, which are linked by disulfide bridges. These fragments are released into the blood stream [23]. Polymorphism in SERA5 has been described in exon II, which corresponds to the amino acid terminal region (47 kD peptide) of the protein-encoding two repeat domains; the octamer repeats (N-terminal = OR domain) and the serine repeats (SR domain). This is characterized by a stretch of serine residues. Studies on the polymorphism of this gene have been carried out in Asia, South America, and West Africa [24], and it was found that only one single OR fragment type (ORI or ORII) appeared in *P. falciparum* malaria-infected participants. In this study, both fragments were observed. This is similar to what was observed in Brazil [17]. While in Brazil, the allele frequencies were similar, in the present study the ORII fragment was more predominant in all the parishes studied in Apac District. In the four parishes, Apac had the highest proportion of the ORII fragment at 57%. This could mean that ORII allele has a role in parasite survival, since it was prevalent among the symptomatic group and also among the < 5 year olds, both groups are vulnerable to severe malaria disease. This might have provided more insight if this analysis was also done among pregnant women, especially primigravidae. This parish also had the highest malaria prevalence among the 5–10-year-olds. In the Brazilian study, there was no relationship between allele type and demographic data and clinical outcomes [17].

In this study there was a strong association with age, clinical outcomes, and parasite density but not with gender (P = 0.68) and bed net use (P = 0.86). The association between ORII fragment with age, parasite density, and clinical outcomes could be an indication that it is a target of immunity. First, the ORII allele was more prevalent in the < 5 years age group (with no clinical immunity and unable to clear parasitaemia) while having a low prevalence in the > 10 years participants (who have developed immunity over time and can clear parasitaemia). Second, the project documented a high prevalence of ORII in participants with a parasitaemia of > 5000 parasites/µL of blood and lower prevalence in subjects with lower parasitaemia. Third, the ORII allele was more prevalent in the symptomatic malaria group by comparison with the asymptomatic group. These observations strongly support the notion that the ORII allele of sera5 is a target of anti-malarial immunity and can provide potential use in sub unit vaccines.

Erythrocyte binding antigen-175 (eba-175) of *P. falciparum* is localized in the merozoites. After the initial contact of the merozoite with the erythrocyte, orientation takes place and the apical end makes contact with the erythrocyte surface [20]. *Eba-175* gene is divided into several domains including three cysteine-rich regions (F1, F2, and F3) [25]. Most *eba-175* sequences have a low level of deduced amino acid polymorphism in its cysteine-rich region II [26, 27]. The central region is highly divergent with dimorphic C and F segments, named after the prototype sequences from the CAMP and FCR-3 isolates, respectively [26]. The *eba-175* alleles/fragments, CAMP and FCR-3, were defined by nested PCR of 178 field isolates in the three study parishes (isolates from Abedi did not amplify). The FCR3 fragment was highest in Apac parish, with prevalence 33%, the CAMP and mixed alleles were highest in Atopi parish with proportions of 10% and 36%, respectively. Overall, the FCR3 fragment had the highest proportion while the CAMP fragment was the least frequent in this study population. This data is consistent with data from Nigeria, Gambia, Gabon, and South Africa in which the FCR3 fragment dominated [20, 28] However, a study in neighboring Sudan showed a different pattern of fragment distribution, with the CAMP fragment being more dominant [28]. This could be explained by the fact that this population represents an ethnically distinct region with lower malaria endemicity [29]. Previous studies did not find any association between parasite density, age, gender, and clinical outcomes and *eba-175* fragment type [20]. In this study, there was association between *eba-175* allele distributions and age, with the FRC3 allele being more prevalent among the < 5 years' age group. The high FCR-3 prevalence in high malaria-endemic areas, especially among the < 5 years old subjects and the low proportion in adults, suggests that FCR3 allele is a target of immune selection pressure. By contrast, the CAMP allele was more prevalent in the > 10 years, an age group known to have acquired immunity against malaria. The CAMP allele was more or less equally distributed in the four parishes as opposed to the FCR-3 allele and mixed alleles that had unequal distribution. This difference in observations between the findings and those of other studies could be explained by random shifts in parasite allele frequencies in different geographical regions [30], as well as the level of malaria endemicity.

CSP is the most abundant protein on the surface of the *P. falciparum* sporozoites. It consists of a sequence comprising a central repeat that is flanked by polymorphic N-terminal and C-terminal non-repeat regions [19], the NANP repeats and single nucleotide polymorphisms (SNPs) in the non-repeat regions. Polymorphisms in the NANP repeats are thought to be extended even in low endemic areas [30]. It has however been documented that SNPs in the non-repeat regions can be stable for a considerable number of years [31]. The *csp* antigen is the primary component of the RTS,S malaria vaccine, which is one of the leading malaria vaccine candidates probably going to be the first malaria vaccine to be deployed in immunization programmes.

The results here represent the first molecular study on the genetic diversity of T-cell epitope regions in the C-terminal portion of *csp* among wild isolates circulating in Apac District, in northern Uganda. Field isolates were amplified by PCR and 61 products were sequenced. Previous studies from moderate to low malaria endemicity settings showed less diversity within this region [31]. Results from this study are consistent with those from other African settings such as the Gambia and Sierra Leone, both high endemicity settings [19], which showed a marked diversity of sequence polymorphisms in the same region of *csp* (Th2R/Th3R). Twenty-six percent (16/61) of the Ugandan sequences had the *P. falciparum* 7G8 reference sequence haplotype and (14/16, 87.5%) of the haplotypes identified were unique to the Ugandan isolates (haplotypes not observed in other studies). One haplotype, which appeared in two samples (2/16, 12.5%) was similar to a haplotype that was previously identified in The Gambia and Sierra Leone [19]. The remaining 43 Ugandan sequences, which did not have the *P. falciparum* 7G8 haplotype at the amino acid level, when aligned with the 7G8 nucleotide sequence showed identities of 97–99%. This confirms the authenticity of the Ugandan sequences and their uniqueness. In this study, the single haplotype that was observed in Asia (Asia type) was not detected [19]. In all, the data underscores the diversity of *P. falciparum* antigens in different geographical regions and the uniqueness of parasite strains from an endemic region in Uganda.

Furthermore, the 3D7 haplotype sequence which is used for the development of the RTS,S malaria vaccine was also not observed in the 61 Ugandan isolates sequenced in this study. This, however, does not rule out the existence of this haplotype (3D7) in Ugandan isolates. It was also observed that at position 326–342 (ThR2) of the *csp* gene, all the Ugandan isolates had a basic amino acid (Lysine, K) while the 3D7 laboratory strain had an acidic amino acid (Glutamic acid, E). This again underscores the divergence of Ugandan *csp* sequences. The absence of the prototype 3D7 sequence in the Apac field isolates could have serious implications on the vaccine accuracy of RTS,S malaria vaccine in this region. This, however, needs to be further investigated, considering the sample size and time from this study. Despite the small sample size, haplotype distribution seemed to take an interesting trend. The majority of haplotypes were observed in the age group under 5 years and also among the symptomatic malaria group. This was not statistically significant. This is comparable to previous data [19], in which a particular haplotype was significantly associated with hospitalized children below 5 years of age. These findings suggest that these haplotypes could be subjected to selective immune selection pressure in the field. These results represent the first study on the genetic diversity of three malaria vaccine candidate antigens in Uganda. Unlike in other studies where the sample was restricted to a narrow age range, this study examined a broader age range and it was able to observe variation in allele distribution and frequency over the age groups. The results of this study show that geographical factors and the local epidemiological situation may affect selection and distribution of specific *P. falciparum* alleles and haplotypes, as was the case for the three selected malaria vaccine candidate antigens, *sera5, eba-*175, and *csp*. For *sera*5, the increased prevalence of ORII allele among individuals with parasite density above 5000 parasites/µL, in symptomatic individuals, and in children < 5 years strongly implicate this allele as a target of immune selection pressure. This observation indicates that the ORII allele stimulates immunity against malaria. This finding further confirms host responses against *sera*5 as a vaccine candidate. The FCR3 allele of *eba-*175 is also an important target of host immunity in the study region. The age-dependent distribution of haplotypes with more haplotypes in the < 5-year-olds, as compared to adults, confirms the role of immunity in haplotype selection. The Ugandan *csp* haplotypes were unique to the population and the 3D7 vaccine type was not observed. The uniqueness of these haplotypes raises concerns about the effectiveness of RTS,S vaccine in this population when deployed, as polymorphisms in the *csp* gene could be a result of immune selection pressure [32]. This however needs to be investigated further.

MOI in the study population was found to be significantly associated with age, parasite density and clinical presentation. These findings are consistent with studies conducted in Sudan which showed that even within the same individual; MOI tends to decrease with age [21]. This could be explained by the effect of acquired immunity which builds with age as a result of multiple exposures to *P. falciparum* infective bites [14].

## Recommendations

It is important to clearly define the genotypes of the parasite population circulating in a region before rolling out a new vaccine. In this regard, the authors recommend the following as important future studies.

1. Country or region-wide genotyping of *P. falciparum* at *csp* locus should be carried out to determine the prevalence of the 3D7 vaccine strain haplotype. This might confirm the uniqueness of the Ugandan strains reported in this study and could provide insights on the future of RTS,S vaccine deployment in this region.
2. Countrywide genotyping of *sera5, eba*-175, and other malaria vaccines candidate antigens genes should also be carried out to identify baseline circulating parasite genotypes before deployment of these vaccines. This could provide insight into future development of more subunit vaccines against malaria.
3. Studies to confirm the immunogenicity of ORII and FCR3 fragments should be undertaken; this will guide development of more effective *sera5* and *eba*-175 subunit vaccines.

## Conclusion

These data suggest that the *eba-175* FCR3 allele, *sera5* ORII allele, and *csp* haplotypes are targets of host immunity and are under immune selection pressure in Apac District. These molecules could provide alternative malaria vaccine candidates as sub-unit vaccines. This work also raises the need for continued monitoring of parasite population dynamics as a tool for monitoring emergence of drug resistance as well as to provide understanding of how these populations are developing mechanisms survive, which could guide both drug and vaccine development. In addition, further studies on evolution of parasite genotypes can also provide insight on development of future insecticides.

## Data Availability

All data generated or analysed during this study are included in this article.
